# The Role of the Yap5 Transcription Factor in Remodeling Gene Expression in Response to Fe Bioavailability

**DOI:** 10.1371/journal.pone.0037434

**Published:** 2012-05-16

**Authors:** Catarina Pimentel, Cristina Vicente, Regina Andrade Menezes, Soraia Caetano, Laura Carreto, Claudina Rodrigues-Pousada

**Affiliations:** 1 Instituto de Tecnologia Química e Biológica, Universidade Nova de Lisboa, Oeiras, Portugal; 2 Department of Biology, Center for Environmental and Marine Studies, Universidade de Aveiro, Aveiro, Portugal; North Carolina State University, United States of America

## Abstract

The budding yeast *Saccharomyces cerevisiae* has developed several mechanisms to avoid either the drastic consequences of iron deprivation or the toxic effects of iron excess. In this work, we analysed the global gene expression changes occurring in yeast cells undergoing iron overload. Several genes directly or indirectly involved in iron homeostasis showed altered expression and the relevance of these changes are discussed. Microarray analyses were also performed to identify new targets of the iron responsive factor Yap5. Besides the iron vacuolar transporter *CCC1*, Yap5 also controls the expression of glutaredoxin *GRX4*, previously known to be involved in the regulation of Aft1 nuclear localization. Consistently, we show that in the absence of Yap5 Aft1 nuclear exclusion is slightly impaired. These studies provide further evidence that cells control iron homeostasis by using multiple pathways.

## Introduction

Iron (Fe) is an essential metal to most forms of life. The function of Fe in biological processes, such as respiration, oxygen transport, DNA synthesis and repair, among others, relies on its capacity to be reversibly oxidized and reduced. However, the same chemical properties that make Fe such a central element for life also make it a strong pro-oxidant that can generate powerful reactive oxygen species (ROS) through Fenton type reactions [1]. Organisms accurately regulate the concentration of Fe levels through regulation of Fe uptake, storage and mobilization [2], [3], [4]. Disturbances of Fe homeostasis affect the pathogenesis of infectious diseases and have severe clinical consequences like Fe-deficiency anemia, Friedreich's ataxia and hereditary haemochromatosis [5], [6], [7].

The budding yeast *Saccharomyces cerevisiae* is able to grow under a wide magnitude of Fe available environments and can survive large fluctuations in Fe bioavailability. Yeast cells respond to Fe deficiency by triggering a complex rearrangement of gene expression that culminates with the activation of Fe transport systems (with the consequent increase of Fe uptake and mobilization from intracellular stores) and the adjustment of metabolism in order to divert iron from Fe-dependent metabolic pathways [8], [9], [10], [11]. The vast majority of these genes are regulated by the Fe-responsive transcription factor, Aft1, and to a lesser extent by its paralogue, Aft2, constituting the iron regulon [2], [3]. Two of the Aft1 targets code for the RNA-binding proteins Cth1 and Cth2, that posttranscriptionally downregulate many mRNAs involved in Fe-dependent processes [9], [12]. Aft1 shuttles between the cytosol and the nucleus, accumulating in the latter under Fe depletion and activating transcription of the Fe regulon [13], [14]. Aft1 activation does not respond directly to cytosolic iron but rather to the production of mitochondrial iron-sulfur clusters via a signaling pathway that requires the activity of the monothiol glutaredoxins Grx3/Grx4 and the regulatory proteins Fra1/Fra2 [13], [15], [16], [17], [18], [19], [20].

Much less is known regarding the response to increased Fe levels in the environment. Unlike humans, but similar to plants, the yeast cell vacuoles function as iron reservoirs. In yeast, iron storage is mediated by Ccc1, a vacuolar transporter that effects the accumulation of iron in the vacuoles [21]. *CCC1* mRNAs are destabilized by Cth2 and Cth1 under iron depleted conditions [9], [12]. In a high-Fe milieu, *CCC1* deletion is lethal [21] and its expression is regulated by Yap5 [22], one of the eight members of the Yap Activator Protein (Yap) family [23].

Herein, we analyzed the transcriptional response of *S. cerevisiae* subjected to high-concentrations of Fe. Microarrays analyses of the *yap5* mutant strain in the presence of Fe excess, allowed us to identify *GRX4* as a Yap5 target. Given the role of Grx4 in Aft1 sub-cellular localization, we analyzed the effect of Yap5 deletion on Aft1 movement to and from the nucleus as a function of cellular iron status. We showed that the absence of Yap5 affects Aft1 localization.

## Results

### Genome-wide transcriptional analysis of *S. cerevisiae* exposed to high iron conditions

Although iron can be toxic, little is known about how iron excess affects metabolic pathways on a global scale in eukaryotic cells.

In order to investigate the response of *S. cerevisiae* to iron excess, we compared the mRNA expression profile of wild-type cells up shifted to Fe-enriched medium (2 mM FeSO4, 20 and 60 min) to cells grown under Fe-adequate conditions (0.044 mg/L, as measured by inductively coupled plasma atomic emission spectroscopy).

**Figure 1:**
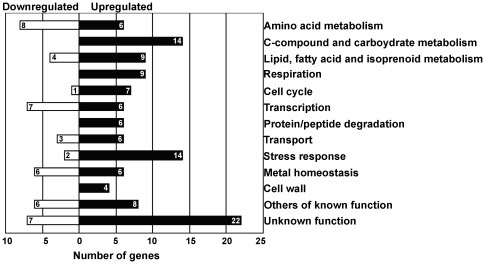
Transcriptional response to Fe overload in yeast. BY4742 wild-type cells were grown to exponential phase in SC medium and challenged with 2 mM of FeSO_4_. RNAs of Fe-treated and untreated cells were isolated and analyzed with DNA microarrays as detailed in *Materials and Methods*. Genes up or downregulated were sorted into functional categories according to MIPS database. Downregulated genes comprised in the Ribosome biogenesis category (n = 48) were not included. A list of all the genes included in each functional category is shown in Table S1.

Iron excess leads to an increase of the mRNA steady-state levels of 117 genes and to the repression of 92 genes (Table S1). Functional categories in the dataset are depicted in Figure 1. The transcript levels of genes included in the category Ribosome biogenesis have shown to be decreased (Table S1), whereas those of Stress response, Protein/peptide degradation, Respiration, Lipid/Fatty acids and Carbohydrate metabolism were found to be increased (Figure 1 and Table S1). These categories are altered whenever the environmental conditions change abruptly and cells rapidly and transiently need to reprogram their profile of gene expression [24], [25].

With respect to changes in the iron regulon, the expression of the Aft1/2 targets *FET3*, *FTR1* and *SMF3* was found to be downregulated (Table 1). *FET3* encodes a plasma membrane multicopper oxidase and *FTR1* codes for an iron permease, constituting a complex that belongs to the high-affinity Fe reductive uptake [2], [3]. *FET3* and *FTR1* genes are coordinately expressed [2], [3] being both transcriptional and posttranslationally regulated by Fe [26]. The gene encoding Smf3, a member of the Nramp family of divalent metal transporters, is involved in pumping iron from the vacuole under iron starvation [2], [3]. Isa1 is a mitochondrial protein, component of the Fe-S complex assembly system (ISC), specifically involved in the maturation of a subset of Fe-S proteins, such as the members of the aconitase superfamily [27] and whose gene is induced in high-Fe (Table 1). *NAR1* that codes for a component of the cytosolic Fe-S protein assembly (CIA) machinery and exhibits a high similarity to bacterial hydrogenases, was shown to be downregulated (Table 1). The expression of *CCC1* gene, that encodes the unique vacuolar iron importer in yeast, is increased upon growth shift to high-Fe (Table 1). Several studies have demonstrated Ccc1 relevance when cells are confronted with Fe-enriched environments [21], [28]. The expression of the monothiol glutaredoxin gene, *GRX4*, is also increased (Table 1). Grx4 together with Grx3 is known to control Aft1 nuclear localization (Ojeda *et al.*, 2006; Pujol-Carrion *et al.*, 2006).

**Table 1:** Genes involved in iron homeostasis with expression altered in Fe excess.

Systematic Name	Gene Name	Fold Change	Description
YMR058w	*FET3*	−6.9	Multicopper oxidase required for high-affinity Fe uptake
YER145c	*FTR1*	−3.3	High affinity Fe permease
YLR034c	*SMF3*	−1.4	NRAMP homolog Fe transporter
YNL240c	*NAR1*	−1.4	Component of the cytosolic iron-sulfur (FeS) protein assembly machinery
YLL027w	*ISA1*	1.5	Protein involved in biogenesis of the iron-sulfur cluster of Fe-S proteins
YLR220w	*CCC1*	2.7	Transporter that mediates vacuolar Fe storage
YER174c	*GRX4*	3.7	Monothiol glutaredoxin

Remarkably, the copper transporter gene, *CTR1*, and metallothioneins *CUP1-1* and *CUP1-2*, three genes involved in copper metabolism are down and upregulated, respectively (Table S1). Copper plays an essential role in reductive Fe uptake, as it is required for Fet3 oxidase activity. Also the levels of Ccs1, a copper chaperone responsible for copper delivery to Sod1 (Cu, Zn superoxide dismutase), previously shown to be elevated in response to Cu deficiency [29], are decreased in high-iron conditions (Figure S1), suggesting that copper metabolism is affected in Fe-enriched environments.

Regarding the oxidative defenses, only the genes encoding the glutaredoxin 2 (*GRX2*) and cytosolic catalase T (*CTT1*) were upregulated upon high-Fe exposure. Grx2 together with glutathione reductase and glutathione play important roles in redox regulation and antioxidant defenses, being the cytosolic catalase T responsible for hydrogen peroxide detoxification [30], [31].

Shakoury-Elizeh *et al.* reported that cells growing in high-Fe medium did not trigger oxidative stress [10]. Consistently, Lin and colleagues showed that the absence of Yap1, the major oxidative stress regulator in yeast, did not influence growth in Fe-enriched medium [32]. Our data suggest that in yeast, oxidative stress solely contributes to iron toxicity (Figure S2), when Ccc1 metal storage is defective and Yap1 is absent, supporting the idea that toxic iron levels should induce cell damage mainly via DNA damage or enzyme mismetallation, as proposed by Lin *et al.* [32].

### Yap5 only partially regulates the expression of *CCC1*

**Figure 2:**
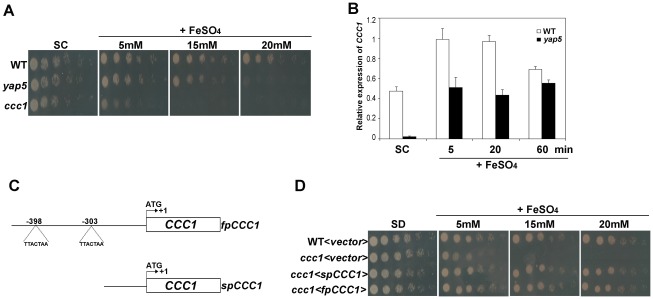
Yap5 is not the only regulator of *CCC1* gene. (A) Exponentially growing cells from wild-type (WT), *yap5* mutant (*yap5*) and *ccc1* mutant (*ccc1*) strains were harvested, serially diluted and spotted onto control SC plates or SC plates containing the designated FeSO_4_ concentrations. (B) WT and *yap5* strains were upshifted to high-Fe medium, by supplementation of SC medium with 2 mM of FeSO4, and harvested at the indicated time-points. The expression of *CCC1* was assessed by qRT-PCR as described in *Materials and Methods*. Values are the mean of biological triplicates ± s.d. (C) Schematic representation of the truncated promoter version of *CCC1* gene (sp*CCC1*). (D) *ccc1* strain was transformed with a plasmid harboring sp*CCC1* gene (*ccc1*<sp*CCC1*>), fpCCC1 gene (*ccc1*<fp*CCC1*>) or the plasmid alone (*ccc1*<*vector*>). Wild-type strain was transformed with the empty plasmid (WT<*vector*>). Exponentially growing cells were harvested, serially diluted and spotted onto control SD plates or SD plates containing 9 mM of FeSO_4_.

In the presence of iron, Yap5 becomes activated, leading to an increased expression of *CCC1* and allowing for re-establishment of iron homeostasis [22]. However, the growth defect exhibited by *ccc1* cells challenged with increased iron concentrations is markedly higher compared to the one displayed by the *yap5* mutant (Figure 2A). While the *ccc1* sensitivity to high-Fe was already observed when cells were grown in 5 mM of FeSO_4_, cells lacking Yap5 only showed a slight impairment of growth above concentrations of 15 mM (Figure 2A). Accordingly, while *CCC1* expression is dramatically reduced in the *yap5* mutant under Fe-adequate conditions, the *CCC1* induction upon a shift to high Fe is not completely compromised, suggesting that *CCC1* is regulated by other metal- or stress-responsive transcription factor. Consistent with Yap5 regulation of *CCC1* expression in Fe-adequate growth medium, we showed that Yap5 is able to activate transcription under this condition (Figure S3).

Next, we examined whether the Yap5-independent levels of *CCC1* expression were enough to cope with toxic Fe concentrations. As such, we generated a construct harboring the *CCC1* coding region and its promoter without the two YREs (Yap Responsive Elements), located at −398 bp and −303 bp upstream from the initiation codon, ATG (Figure 2C). The construct designated by spCCC1 was then used to transform the *ccc1* mutant cells and growth was assayed in plates containing Fe. We verified that *ccc1* cells carrying the truncated version of *CCC1* promoter were able to rescue *ccc1* growth phenotype (Figure 2D). These data indicate that the YRE elements are not the only cis-elements implicated in the transcriptional response of *CCC1* upon exposure to iron excess and corroborate the hypothesis that another not yet identified factor may also mediating *CCC1* gene expression.

### Yap5 targets under high-iron conditions

Recently it was described that Yap5 regulates the expression of *TYW1* gene that codes for an iron-sulfur cluster enzyme that participates in the synthesis of wybutosine modified t-RNA, whose overexpression alleviates the *ccc1* high Fe sensitivity [33].

Aiming at identifying other Fe-dependent targets of Yap5, we characterized the global changes in the transcriptome of *yap5* mutant cells in the presence of high levels of iron. DNA microarrays analyses were conducted by comparing RNAs isolated from the *yap5* mutant vs. wild-type strains subjected to high-Fe concentrations for 20 and 60 min (Tables S2, S3). The total number of genes with altered expression in a *yap5* strain, upon growth shift to high-Fe medium for 20 or 60 minutes, was 55 and 199, respectively.

**Figure 3:**
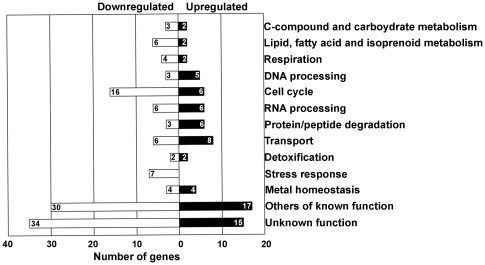
Functional categories of genes differentially expressed in *yap5* mutant cells. BY4742 wild-type and *yap5* mutant strains were grown to exponential phase in SC medium and challenged with 2 mM of FeSO_4_ for 60 min. RNA of Fe-treated cells was isolated and analyzed with DNA microarrays as detailed in *Materials and Methods*. Genes up- or downregulated were sorted into functional categories according to MIPS database. Dubious open reading frame unlikely to encode a protein (100) were not considered. A list of all the genes included in each functional category is shown in Table S3.

Genes differentially expressed were sorted into functional categories according to MIPS. Our data showed that some categories were significantly more represented in *yap5* mutant compared to wild-type cells (Figure 3). Interestingly, genes associated with Cell Cycle category were downregulated. It has been described that SBF (Swi4-Swi6 cell cycle box binding factor) binds the promoter of Yap5 [34], and this might explain the downregulation of genes involved in cell cycle.

We searched for genes direct or indirectly involved in iron homeostasis, whose expression is altered under the tested experimental conditions. We found out that in addition to *CCC1* and *TYW1*, Yap5 regulates the expression of *GRX4* and *FET3* genes (Table 2). We focused our studies on GRX4, which encodes a glutaredoxin known to be involved in the regulation of Aft1 localization [19], [20].

**Table 2:** Genes involved in iron homeostasis whose expression is altered in the *yap5* mutant.

Systematic Name	Gene Name	FC* 20 min	FC* 60 min	Description
YMR058w	*FET3*	1.5	2.4	Multicopper oxidase required for high-affinity Fe uptake
YLR220w	*CCC1*	−2.9	−1.7	Transporter that mediates vacuolar Fe storage
YER174c	*GRX4*	−2.7	−1.9	Monothiol glutaredoxin
YPL207w	*TYW1*	-	−1.5	Protein required for the synthesis of wybutosine

* FC-Fold Change.

### *GRX4* is transcriptionally regulated by Yap5

**Figure 4:**
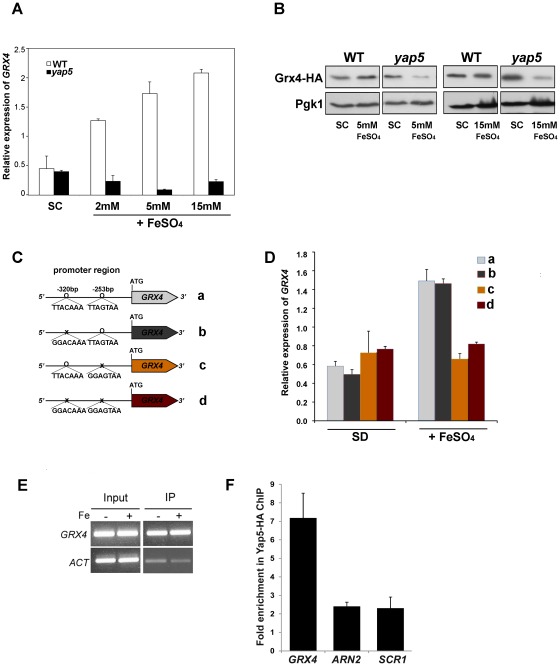
*GRX4* gene expression is dependent on Yap5. (A) Exponentially growing cells from wild-type (WT) and *yap5* mutant (*yap5*) strains were upshifted to high-Fe medium, by supplementation of SC medium with the indicated FeSO_4_ concentrations, and harvested at the indicated time-points. The expression of *GRX4* was assessed by qRT-PCR as described in *Materials and Methods*. Values are the mean of biological triplicates ± s.d. (B) WT and *yap5* strains were transformed with a plasmid carrying *GRX4*-HA, treated with 5 and 15 mM of FeSO_4_ for 20 min and analyzed by Western blot with an anti-HA antibody. Pgk1 protein levels were used as loading control. (C) Representation of the *GRX4* constructs without (a) or with (b,c,d) mutations in the YREs. (D) Mutant *grx4* cells expressing the constructs depicted in (C) were grown under Fe-adequate or Fe overload (5 mM FeSO_4_) conditions, and the expression of *GRX4* was assessed by qRT-PCR as described in *Materials and Methods*. Values are the mean of biological triplicates ± s.d. (E) *yap5* cells were transformed with HA-tagged Yap5 and grown in SD medium not supplemented (−Fe) or supplemented (+Fe) for 15 min with 2 mM of FeSO_4_, before being processed for ChIP. ChIP analysis was performed using probes specific for *GRX4*. (F) ChIP analyses combined with qRT-PCR, were used to determine the fold enrichment of *GRX4*, *SCR1* and *ARN2*. The sequence enrichment in the ChIP (i.e. IP/IN) was normalized using the *ACT* gene as a reference.

To investigate the transcriptional regulation of *GRX4* by Yap5, we followed by qRT-PCR Grx4 mRNA levels in the presence of several iron concentrations, in the wild-type and mutant *yap5* strains (Figure 4A). Upon growth shift to high Fe, there was an increase of *GRX4* mRNA levels that was absent in the *yap5* mutant. We also monitored the protein expression *of a* HA-tagged version of Grx4 driven by its native promoter and observed that according to gene expression data, protein levels were decreased in the mutant strain (Figure 4B).

Using YEASTRACT [35], we found that *GRX4* possesses in its promoter region 2 potential YREs, placed at −253 bp and −320 bp from the ATG (Figure 4C). To investigate whether both YREs are functional, we cloned *GRX4* gene and its promoter in a centromeric plasmid and mutated each or both YRE(s) (Figure 4C). The resulting constructs were used to transform the *grx4* mutant and *GRX4* expression was monitored by qRT-PCR (Figure 4D). In the presence of high Fe, there was a remarkable decrease in the expression of *GRX4* when the YRE located at −253 bp or both YREs were mutated (Figure 4D). Mutation of the −320 bp YRE had no effect in *GRX4* expression, suggesting that the YRE closer (−253 bp) to ATG is necessary and sufficient for Yap5 iron-regulated expression of *GRX4*.

We carried out chromatin immunoprecipitation (ChiP) analyses, using a HA-tagged version of Yap5, and detected a significant binding of Yap5 to *GRX4* promoter even in the absence of Fe (Figure 4E). As depicted in Figure 4E, after immunoprecipitation of chromatin bound to Yap5-HA (IP), an enrichment of *GRX4* promoter harboring the YRE located at −253 bp was observed, as compared to the promoter of Actin gene (ACT) that served as a negative control of binding. The same pattern of transcriptional regulation was observed by others regarding Yap5 regulation of *CCC1* expression [22].

Yap5 binding specificity to *GRX4*, in the Yap5-HA ChiP, was further confirmed by comparing the fold enrichment of *GRX4* with two non-harboring YREs genes-*SCR1* and *ARN2-* coding for an ncRNA and a siderophore transporter, respectively (Figure 4F). *GRX4* exhibits significant threefold enrichment compared to *SCR1* and *ARN2* in the Yap5-HA ChiP, as illustrated in Figure 4F.

Our studies corroborate a previous genome-wide location analysis in which is shown Yap5 bound to *GRX4* promoter region [36].

Together these data suggest that the binding of Yap5 to *GRX4* is Fe-independent and that Yap5 is directly regulating the iron-dependent transcription of *GRX4* gene.

### Aft1 localization is affected in the yap5 strain under Fe-adequate and Fe overload growth conditions

Because *GRX4* transcription is dependent on Yap5 (Figure 4) and given that Grx4 together with Grx3 controls Aft1 nuclear localization, we next questioned whether Aft1 localization is affected in the *yap5* mutant strain.

**Figure 5:**
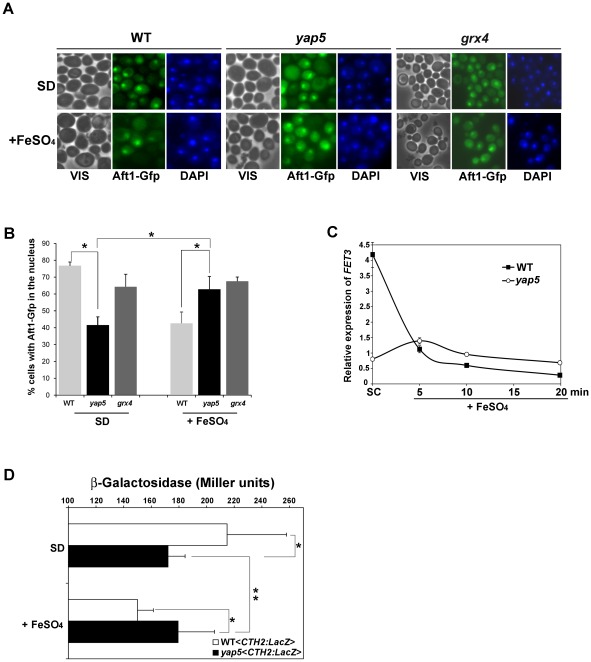
Aft1 localization is affected in the absence of Yap5. (A) Wild-type (WT) *yap5* (*yap5*) *grx4* (*grx4*) mutant strains were transformed with a high-copy *AFT1-GFP* plasmid. Exponentially growing cells in SD medium were untreated (SD) or treated with 2 mM of FeSO_4_ for 30 min. GFP-Aft1 fusion protein was visualized by fluorescence microscopy. (B) Cells with nuclear fluorescence were counted as detailed in *Materials and Methods*. Approximately 100 cells were analyzed per condition. Values are the mean of two biological duplicates (200 cells) ± s.d. (C) Exponentially growing cells from wild-type (WT) and *yap5* mutant (*yap5*) strains were upshifted to high-Fe medium, by supplementation of SC medium with 2 mM of Fe SO4, and harvested at the indicated time-points. RNAs were isolated and the expression of *FET3* was assessed by qRT-PCR as described in *Materials and Methods*. Values are the mean of biological triplicates ± s.d. (D) WT and *yap5* strains were transformed with the plasmid pCM64-*CTH2*-FeRE-*CYC1*-LacZ (*CTH2:LacZ*). Cells were grown exponentially in SD medium or in Sd medium supplemented with 2 mM of FeSO_4_, for 30 min (+FeSO_4_) and ß-galactosidase activity was assayed as detailed in *Materials and Methods*. Values are the mean of biological decaplicates ± s.d. * P<0.01 **P<0.05.

We first examined the localization of a plasmid overexpressing Aft1 fused to GFP [20] in the wild-type, *yap5* and *grx4* mutant strains, by *in vivo* fluorescence microscopy. Cells exhibiting nuclear fluorescence were counted before (SD medium) and after (+FeSO_4_) Fe addition to the culture. Under Fe-adequate condition (SD) *yap5* was the strain that exhibited less Aft1 in the nucleus (Figure 5A and 5B). On the contrary, when cells were shifted to Fe-enriched medium (+FeSO_4_), there was more cells with Aft1 in the nucleus in the *yap5* mutant compared to the isogenic parent strain (Figure 5A and 5B). The latter effect might be a consequence of the Fe-dependent Yap5 regulation of *GRX4* (Figure 4A), since *grx4* mutant cells also exhibited a higher content of Aft1 in the nucleus upon Fe addition to the culture (Figure 5A and 5B).

To further confirm these results, we assayed the dependence on Yap5 of the Aft1 target *FET3* through qRT-PCR (Figure 5C). Accordingly to our previous observations on Aft1 localization, we noticed that *FET3* expression is repressed in the *yap5* mutant under Fe-adequacy, whereas under Fe-overload *FET3* is slightly induced in the mutant (Figure 5C).

In addition, we used another indirect approach to test the influence of Yap5 in Aft1 localization. Wild-type and *yap5* strains were transformed with a plasmid (*CTH2-LacZ*) containing the consensus Aft1 binding sequences of *CTH2* promoter fused to a *lacZ* reporter [12]. *CTH2* and *CTH1* are gene targets of Aft1, both expressed in iron deficiency. Corroborating the above assays, we observed that under Fe adequacy (SD) the ß-galactosidase activity was higher in the wild-type compared to the mutant strain, but upon a growth-shift to high Fe *yap5* mutant exhibited the highest ß-galactosidase expression (Figure 5D). Similar experiments with the same construct carrying a mutation in the Aft1 binding site [12] did not exhibit significant ß-galactosidase activity.

Together these data indicate that Yap5 interferes with Aft1 nuclear localization under Fe adequacy and Fe overload, in opposite ways. Under Fe-adequate conditions there is less Aft1 in the nucleus in the *yap5* mutant, whereas under high Fe conditions there is more Aft1 in the nucleus of the mutant cells.

## Discussion

Disruption of the iron homeostasis dynamic process originates significant damage in cells. Therefore, organisms and yeast cells in particular have evolved sophisticated mechanisms that, on one hand avoid the drastic consequences of iron scarcity and, on the other hand circumvent the toxic effects of iron overload. It is relevant to understand how cells cope with high Fe levels, since hemachromatosis caused by Fe overload is one of the most common human genetic diseases.

The work here reported shows that, as transcriptional primary response to iron excess, *S. cerevisiae* represses genes involved in iron uptake, *FET3* and *FTR1*, as well as in iron mobilization from the vacuole, *SMF3*; it also induces genes implicated in Fe storage, *CCC1*, and in Aft1 export from the nucleus, *GRX4*. Apparently, the alteration in the expression pattern of these genes is enough to control the intracellular iron concentration that would lead to Fe-induced toxicity. *CCC1* induction as well as *FET3* and *FTR1* repression, when in the presence of high-Fe, have been also reported by other authors [21], [22], [26]. Surprisingly, only three genes of the Fe-regulon were found to be downregulated (see Table 1, *FET3*, *FTR1* and *SMF3*). Because under iron overload conditions Aft1 is exported from the nucleus, one would expect to see a greater number of Aft1-dependent genes being downregulated. Yun *et al.* noticed that Aft1-dependent transcription is repressed in Fe-sufficient medium [37]. Furthermore, under these conditions, RNAse III Rnt1-mediated RNA surveillance is required to prevent iron toxicity [38]. Rnt1 degrades several Aft1-dependent targets, but not *FET3* or *FTR1* mRNAs; this may explain why we did not detect other Aft1 targets.

The novelty of our analysis is the induction of *GRX4* in a Fe-dependent manner. The physiological function of Grx4/3 has been extensively studied and its role in Aft1 activity inhibition was proposed to be due to the sequester of this transcription factor in the cytosol under iron overload conditions [19], [20]. Hence, it is likely that cells increase Grx4 levels when facing high-Fe concentrations in order to guarantee that Aft1 is efficiently removed from the nucleus. Grx4/3 may also participate in intracellular iron trafficking, as the double mutant *grx4grx3* elicits severe defects in the maturation of cellular Fe-S proteins, heme-containing and di-iron enzymes, despite the constitutive Aft1 activation and the consequent cytosolic iron accumulation [39], [40]. Shakoury-Elizeh *et al.* observed that under high-Fe pathways involving biotin biosynthesis and nitrogen assimilation via glutamate synthase (Glt1) were activated [11]. These pathways requiring Fe-dependent enzymes may possibly lead to the increase of Grx4 levels that correlates with the shift to a most Fe-consuming metabolism. Grx3 and Grx4 bind a Fe-S center together with gluthatione which is crucial for their function [39], [40], [41]. Therefore another possibility is that Grx4, itself, may buffer the increased cytosolic iron concentrations under such conditions.

Under iron overload conditions, the transcription factor Yap5 is activated and increases the expression of *CCC1* that transports iron into the vacuole, leading to the consequent decrease of the cytosolic iron pool [22]. We have now shown that although Yap5 does indeed regulate *CCC1* transcription, another yet unidentified Fe-responsive factor drives the expression of *CCC1* up to sufficient levels to overcome Fe toxicity. The putative existence of another Fe-responsive factor could justify the almost-normal growth displayed by the *yap5* strain in high-Fe medium, as well as the rescue of the poor-growth phenotype of the *ccc1* strain by a plasmid harboring the *CCC1* gene without YREs (Figure 2). Furthermore, we showed that, under these conditions, in addition to *CCC1*, Yap5 is also regulating the expression of *GRX4* (Figure 4). Consistently with Grx4 dependence on Yap5, we demonstrated, using different approaches, that Yap5 affects Aft1 localization under Fe overload: *yap5* cells have more Aft1 in the nucleus and consequently exhibit an upregulation of *FET3* (Figure 5). We cannot however exclude the possibility that Yap5 might as well play a role in iron trafficking, or in buffering iron excess, due to the recent findings on the role of glutaredoxins Grx3/Grx4 in iron metabolism [39], [40].

Moreover, we demonstrated that Yap5 also plays a role under Fe-adequate growth environments, as in such condition, Yap5 is the major regulator of *CCC1* (Figure 2A). As such, in the *yap5* mutant strain, *CCC1* levels are severely compromised, leading to an increase of cytosolic iron with the consequent inhibition of Aft1 (that localizes mainly in the cytosol) and the concomitant downregulation of its target *FET3* (Figure 5).

**Figure 6:**
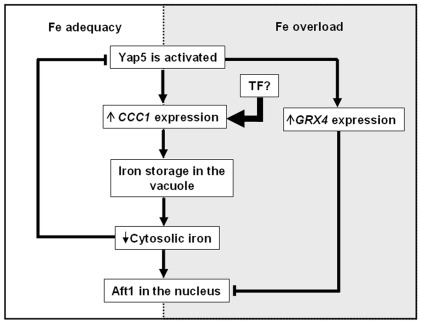
Model for the hypothetical role of Yap5 in iron metabolism. Under Fe-adequate growth conditions Yap5 is active and the main regulator of *CCC1* gene expression. The continuous Fe storage will end up with cytoslic Fe depletion and the consequent activation of Aft1 in order to re-establish Fe homeostasis. Under Fe overload, another factor is regulating *CCC1* gene expression, bypassing Yap5 regulation. In addition Yap5 transactivation potential is higher and therefore it is able to induce *GRX4* gene that together with Grx3 will inhibit Aft1 activity by promoting its nuclear export to the cytoplasm.

In light of the data described herein, we propose that under Fe-adequacy Yap5 is activated leading to the upregulation of *CCC1* with the consequent accumulation of Fe in the vacuole and deprivation of the cytosolic iron pool. As a result, Aft1 translocates to the nucleus and upregulates iron uptake genes. Under Fe overload conditions, Yap5 transactivation potential increases leading to the upregulation of *GRX4* gene and thus inhibiting Aft1 nuclear localization. Simultaneously, it seems that another iron responsive factor is activated, contributing to the *CCC1* induction and most probably bypassing Yap5 regulation (Figure 6). Further work is in progress in order to clarify the mechanism that controls the Yap5-independent regulation of *CCC1*.

## Materials and Methods

### Strains, growth conditions and sensitivity analysis

Most of the experiments were performed with the wild-type BY4742 (*MAT alpha*; *his3*Δ*1*; *leu2*Δ*0*; *lys2*Δ*0*) and its isogenic derivates. The *yap1*, *yap*, *grx4* and *ccc1* strains were purchased from Euroscarf. To produce the double mutant strain *yap1ccc1*(*MAT alpha*; *leu2*Δ*0*; *lys2*Δ*0*; *ura3*Δ*0; yap1::HIS*, *YLR220W::kanMX*), the complete coding region of *YAP1* gene was deleted by the microhomology PCR method [42] from the *ccc1* strain. Deletion was confirmed by PCR analysis of genomic DNA using upstream and downstream *YAP1* specific primers. EGY48 (*Mat alpha ura3-52 his3 LEXA_op(x6)_-Leu2* <pSH18-34 *URA3* 2 µ> was used for transactivation potential assay. Strains were grown in synthetic media (SC: 0.67% ammonium sulphate-yeast nitrogen base without amino acids [Difco], 2% glucose, supplemented with the appropriate selective amino acids) or SC lacking specific requirements (SD). Phenotypic growth assays were carried out by spotting 5 µl of early exponential phase cultures (A_600_ = 0.4) sequentially diluted (approximately 5×10^3^ to 10 cells) in medium containing up to 20 mM of FeSO_4_. Growth was recorded after 2 days at 30°C. Anaerobiosis assays were performed with cultures transferred to a glove box, for at least 24 h, where the O_2_ concentration was kept below 1 ppm. The strains were grown in liquid SD until early exponential phase and phenotypic growth assays were performed as described above. *Escherichia coli* strain XL1-Blue *recA1 endA1 gyrA96 thi-1 hsdR17 supE44 relA1 lac* [F′*proAB lacIqZDM15 Tn10* (Tetr)] (Stratagene) was used as the host for routine cloning purposes. Standard methods were used for genetic analysis, cloning and transformation.

### Plasmids

The *fpCCC1* plasmid was generated by blunt cloning into the vector pRS416 (Stratagene) the *CCC1* gene amplified by PCR using BY4742 genomic DNA as template and the primers 5′TACAGGACCAAACCCCTCAG3′ and 5′GGCCTCTTGGCAATAGAAAA3′. The construct *spCCC1* was obtained by digesting *fpCCC1* with *Kpn*I (Fermentas).

Yap5-HA contains a hemagglutinin (HA) epitope inserted immediately upstream the stop codon, and is expressed from the centromeric pRS416 plasmid. The following three-step PCR strategy was used to HA-tagged Yap5: *YAP5* promoter region and coding sequence were amplified using the primers CAAAACTGTGGTGTTAG/GTATTAAGAAGTTCTCTCTCTTCAAGCGTAATCTGGAACATCGTATGGGTACATGTGGATGATGGACCGG (the latter contained the HA sequence); a second PCR, using the pair CCGGTCCATCATCCACATGTACCCATACGATGTTCCAGATTACGCTTGAAGAGAGAGAACTTCTTAATAC/GAATGTATAGGCATAGTAAG, amplified the *YAP5* terminator; PCR products were than purified and finally the first and second primers of the first and second PCR reactions, respectively, were used to fuse by means of PCR both products. A similar strategy was used to produce Grx4-HA, using the primer combination AGCAAGGACTGGGACCCTAT/CATTACAAAATAAGGCTTAAGCGTAATCTGGAACATCGTATGGGTACATCTGTAGAGCATGTTGGAAATATTC (first PCR) and GAATATTTCCAACATGCTCTACAGATGTACCCATACGATGTTCCAGATTACGCTTAAGCCTTATTTTGTAATG/ACGAGAAAACCCCAGGAAAT (second PCR). The final PCR product was blunt cloned into the pRS416 plasmid.

All PCR amplifications were performed with Phusion High-Fidelity DNA Polymerase (Finnzymes) and inserts were sequenced.

The plasmid Yap5-lexA used in this work has been described previously [43].

Plasmids containing consensus (pCM64-*CTH2*-FeRE-*CYC1*-LacZ) or mutant (pCM64-*CTH2*-FeRE-*CYC1*-LacZ M3) Aft1 binding sequences from *CTH2* promoter fused to the *CYC1* minimal promoter-LacZ reporter [12] were a gift from Dr. D. Thiele.

AFT1-GFP plasmid [20] was a gift from Dr. M. Toledano.

*GRX4* gene and its promoter were amplified by PCR with the primers CTTAAGCCTTATTTTGTAATG/ACGAGAAAACCCCAGGAAAT and blunt cloned into pRS416. The first upstream YRE in GRX4 promoter region was mutated by site-directed mutagenesis as detailed in [44] with two primers: ggaatactttggacaaacttgg/ccaagtttgtccaaagtattcc. The second YRE in GRX4 promoter region was mutated using the same strategy, but using the primer pair: tcgtgaaattggagtaagagcg/cgcgctcttactccaatttcacga.

### Microarray and qRT-PCR analyses

DNA microarray analysis was carried out using *in-house* spotted DNA-microarrays *of Saccharomyces cerevisiae*, and standard protocols of public domain software used by the NFDM.

For transcript profiling, total RNA from early log-phase cultures, either untreated or exposed to 2 mM of FeSO_4_, was purified using the RNAeasy kit (QIAGEN), followed by the RNA clean up procedure. Forty micrograms of RNA were used to generate labeled cDNA, which were hybridized on the DNA arrays. Images of the microarray hybridizations were acquired using the Agilent G2565AA microarray scanner. The fluorescence intensities were quantified with QuantArray v3.0 software (PerkinElmer). Using BRB-ArrayTools v3.4.0 software [45], manually flagged bad spots were eliminated and the local background was subtracted before averaging the replicate features on the array. Log_2_ intensity ratios (M values) were then Median normalized to correct for differences in labeling efficiency between samples.

The relative hybridization signal of each ORF was derived form the average of two dye-swap hybridizations performed for each strain. The normalized log_2_ ratio (M value) was considered as a measure of the relative abundance of each ORF relatively to that of the reference strain BY4742. Deviations from the 1∶1 hybridization ratio were taken as indicative of changes in gene expression.

T-test analyses, with p value<0.05 were performed using the algorithm implemented in MeV from TM4 software [46]. The individual hybridizations were used as the input data, in a total of two dye-swap hybridizations for each strain. Functional annotations and GO terms association was done following the *Saccharomyces* Genome Database (SGD) annotations (http://www.yeastgenome.org/).

The array design, spotting protocol, raw data and pre-processed data from all hybridizations were submitted to the ArrayExpress Database and can be accessed using the accession number E-MEXP-3193.

Searches for putative Yap binding sites were carried out using the YEASTRACT database [35]. Gene clustering was performed according to the Munich Information Center for Protein Sequences database (MIPS) functional catalogue (http://mips.helmholtz-muenchen.de/proj/funcatDB/).

For qRT-PCR experiments, RNA was extracted from early log-phase cultures that were either untreated or exposed to the indicated FeSO_4_ concentrations and harvested at the indicated time points. DNA was removed by on-column DNAse I digestion (RNase-Free DNase Set; Qiagen). Total RNA (1 µg) was reverse transcribed with Transcriptor Reverse Transcriptase (Roche Diagnostics). Gene primer sequences used in the analyses were as follows: *CCC1:* AACGCAGTGGTGACCCTTAT/TCCTGCGCTGAATTTCTACC, *GRX4:* TGCCTCACTAGCGAACAATG/AGGTTCTGATGGGCTTCCTT and *FET3:* ACGGTGTGAATTACGCCTTC/TTGGAAAGCGTGACCATGTA. qRT-PCR reactions were performed in the Light Cycler 1.5 Real-Time PCR System (Roche), using Light Cycler Fast Start DNA Master SYBR Green I (Roche). Relative standard curves were constructed for each gene, using triplicate serial dilutions of cDNA. The relative expression of the genes was calculated by the relative quantification method with efficiency correction, using the LightCycler Software 4.1. Actin gene was used as a reference gene. All assays were made in triplicate.

### ChIP analysis

Chromatin immunoprecipitation (ChIP) assays were carried out as previously described [47]. 50 ml of cell cultures harvested at OD600 1±0.1 were fixed with 1% formaldehyde for 30 min at room temperature, with occasional agitation. The cross-linking was stopped by the addition of glycine to a final concentration of 340 mM. Cells were collected by centrifugation and were disrupted with a FastPrep®-24 instrument (MP Biomedical) in lysis buffer (50 mM HEPES-KOH pH 7.5, 140 mM NaCl, 1 mM EDTA, 1% Triton X-100 and 0.1% Na-deoxycholate) containing a protease inhibitor cocktail (Roche) and phenylmethylsulfonyl fluoride (PMSF) to a final concentration of 1 mM. The cell extracts were collected and subjected to sonication to yield DNA fragments in a size range between 100 to 1,000 bp with an average of 500 bp. The cross-linked chromatin was separated from the insoluble debris by centrifugation for 10 min at 10000*g* at 4°C.HA-tagged Yap5 was immunoprecipitated by incubating the cross-linked chromatin with the HA-antibody prebound to 50 µl of Dynabeads Pan Mouse IgG (Invitrogen) for 16 h at 4°C. Immune complexes were washed twice in lysis buffer containing 360 mM NaCl, once in wash buffer (10 mMTris-HCl pH 8.0, 250 mM LiCl, 1 mM EDTA, 0.5% NP-40 and 0.5% Na-deoxycholate) and once in TE. Immunoprecipitated proteins were eluted from the beads by heating the samples for 20 min at 65°C in elution buffer (50 mM Tris-HCl pH 8.0, 10 mM EDTA and 0.5% SDS), with agitation at 12000 rpm, and fixation was reversed by heating the eluates for 16 h at 65°C. Aliquots of total chromatin input (IN) and immunoprecipitated (IP) chromatin were simultaneously processed for subsequent normalization. After treatment of samples with proteinase K and RNAse A the DNA was purified, using the DNA Clean & Concentrator system (ZYMO RESEARCH), and was eluted in 30 µl TE. Quantification of specific DNA targets (*GRX4*, *ACT1* and *SCR1*) in the IN and IP samples was performed by real-time PCR. A standard curve, generated with a dilution series of the IP sample, was used to assess the PCR efficiency and the relative enrichment of a specific locus in the immunoprecipitate was determined using the ΔΔCT method through the calculation of log2 (IP/IN). The primer sequences were the following: *SCR1*:cgtctctctgtctggtgcgg/atcccggccgcctccatcac; *ARN2*: aggtatgctgctggagctgt/gagggccatgaaggtatcaa; *GRX4*: ggcaaaagggtctgaaaattt/ctaaatatcaaccagtcttcag. The latter primer pair was used to amplify the region of the *GRX4* promoter flanking the YRE located at −253 bp from the ATG. *GRX4* primers were designed in order to exclude the promoter region harboring the YRE located at −320 bp.

### Measurements of ß-galactosidase activity

The strain EGY48 carrying pSH18-34 (2 µ plasmid carrying a *lacZ* reporter gene under the control of eight *lexA* operators) was transformed with Yap5-LexA. BY4742 and its isogenic strain *yap5* were transformed with the plasmids pCM64-*CTH2*-FeRE-*CYC1*-LacZ and pCM64-*CTH2*-FeRE-*CYC1*-LacZ M3. Cells were grown in SD liquid medium to early log phase, in the presence of 100 µM of BPS (Bathophenatrolinedisulfonic acid disodium salt hydrate, Alfa Aesar) or exponentially grown in SD and challenged with 2 mM of FeSO_4_ and harvested after 30 min. Relative ß-galactosidase activity was monitored as in [48]. Enzyme activity was assayed by following the degradation of the colorimetric substrate ONPG (o-nitrophenyl-b-D-galactopyraniside) at A_420_ and normalized against total protein concentration. The results are the average of at least three biological replicates (n = 3).

### Fluorescence microscopy

BY4742 and *yap5* strains transformed with a plasmid containing *AFT1* fused to *yEGFP3* [20], were grown to early log phase and induced with 2 mM of FeSO_4_ for 30 minutes. 4.6-Diamino-2-phenylindole (DAPI) was added as a DNA marker at a final concentration of 5 µg/ml, 7 min before microscopy. After washing with phosphate-buffered saline (PBS), cells were resuspended in DABCO solution (75% (v/v) glycerol, 0.25× PBS and 200 mM diazabicyclooctane, Sigma–Aldrich). GFP signals were analyzed in living cells with a LEICA DMRXA fluorescent microscope equipped with a Roper Scientific Micro-Max cooled CCD camera and MetaMorph software (Universal Imaging, Inc.) was used to count cells. Ate least 100 cells/strain/condition were counted.

### Immunoblot Assays

Western blot analyses were performed using early exponential phase cells, challenged with 5 mM or 15 mM of FeSO_4_ and harvested at the indicated time-points.

Protein extracts were generated from cell cultures using cell lysis buffer (50 mM HEPES, pH 7.5, 1 mM EDTA, 100 mM KCl, 10% glycerol, 0.1% NP40) supplemented with protease inhibitors (Roche). Protein concentrations were determined using the Bradford assay and 80–100 µg of protein was resolved by SDS-PAGE, and transferred to a nitrocellulose membrane. Protein levels were detected using Anti-HA-Peroxidase, High Affinity from rat IgG1 (Roche) and Anti-α-Sba1 or Anti-α-Pgk1. Sba1 and Pgk1 were used as loading controls [49].

## Supporting Information

Table S1 Genes whose mRNA steady-state levels are altered in Fe excess (2 mM FeSO_4_).
(PDF)

Table S2 Genes dependent on Yap5 whose mRNA steady-state levels are altered upon cells incubation with 2 mM of FeSO_4_ for 20 min.
(PDF)

Table S3 Genes dependent on Yap5 whose mRNA steady-state levels are altered upon cells incubation with2 mM of FeSO_4_ for 60 min.
(PDF)

Figure S1 **Copper metabolism in yeast is affected upon growth shift to high-Fe medium.** BY4742 wild-type cells were transformed with a plasmid containing *CCS1* (copper chaperone for Cu/Zn superoxide dismutase) HA-tagged and exponentially grown in SD medium. (A) Cells were treated with 2 mM of FeSO_4_, harvested at the indicated time-points and examined by Western blot with an anti-HA antibody. (B) Ccs1-HA response to high-Cu (9 mM CuSO_4_, 60 min) was monitored by Western blot and served as Ccs1-HA functional control. Sba1 protein levels were used as loading control.
(PDF)

Figure S2 **In the absence of Ccc1, Yap1 is required for cells to overcome Fe-induced oxidative stress.** Exponentially growing cells from wild-type (BY4742), *yap1*, *yap1ccc1* and *ccc1* strains were harvested, serially diluted and spotted onto control SC plates or SC plates containing the indicated FeSO_4_ concentrations under (A) aerobiosis and (B) anaerobiosis.
(PDF)

Figure S3 **Yap5 transactivation potential in different media used in this work.** (**B**) The transactivation potential of Yap5 in SD medium not supplemented (SD) or supplemented with 100 µM of BPS (SD-Fe), or 2 mM of FeSO_4_ (SD+Fe), was assayed. EGY48 strain carrying pSH18-34 (a plasmid carrying a *lacZ* reporter gene) was transformed with Yap5-LexA and ß-galactosidase activity was monitored as described in *Experimental procedures*. Values are the mean of triplicate samples of the same experiment ± s.d.
(PDF)
