# Supplementary material for: The Role of the Yap5 Transcription Factor in Remodeling Gene Expression in Response to Fe Bioavailability
Source: PLoS One. 2012 May 16;7(5):e37434. doi: 10.1371/journal.pone.0037434 (PMC3353947; doi:10.1371/journal.pone.0037434)
Supplement: Table S1 — Genes whose mRNA steady-state levels are altered in Fe excess (2 mM FeSO4). (PDF) [file pone.0037434.s001.pdf]

Table S1. Genes whose mRNA steady-state levels are altered in Fe excess (2mM FeSO4)

Systematic Name	Gene Name	Fold Change	Description
**METABOLISM**			
**Amino acid metabolism**			
YJR109C	CPA2	-2.4	Large subunit of carbamoyl phosphate synthetase
YOL058W	ARG1	-2.2	Arginosuccinate synthetase
YDL182W	LYS20	-2.1	Homocitrate synthase isozyme
YOR302W		-1.9	CPA1 uORF
YOR303W	CPA1	-1.8	Small subunit of carbamoyl phosphate synthetase
YER069W	ARG5,6	-1.7	Protein that is processed in the mitochondrion to yield acetylglutamate kinase and N-acetyl-gamma-gl
YHR018C	ARG4	-1.6	Argininosuccinate lyase
YJL088W	ARG3	-1.6	Ornithine carbamoyltransferase
YGR055W	MUP1	1.4	High affinity methionine permease
YMR020W	FMS1	1.5	Polyamine oxidase
YIL042C	PKP1	1.5	Mitochondrial protein kinase
YER091C	MET6	1.7	Cobalamin-independent methionine synthase
YDR502C	SAM2	1.9	S-adenosylmethionine synthetase
YBR208C	DUR1,2	2.3	Urea amidolyase
**C-Compound and Carbohydrate metabolism**			
YLR345W		1.5	Similar to 6-phosphofructo-2-kinase
YOR374W	ALD4	1.5	Mitochondrial aldehyde dehydrogenase
YDL124W		1.5	NADPH-dependent alpha-keto amide reductase
YNL134C		1.6	Putative protein of unknown function with similarity to dehydrogenases
YLR251W	SYM1	1.6	Protein required for ethanol metabolism
YOR347C	PYK2	1.6	Pyruvate kinase that appears to be modulated by phosphorylation
YOR344C	TYE7	1.6	Serine-rich protein that contains a basic-helix-loop-helix (bHLH) DNA binding motif
YNL274C	GOR1	1.6	Glyoxylate reductase
YLR258W	GSY2	1.8	Glycogen synthase
YPR160W	GPH1	1.8	Non-essential glycogen phosphorylase
YCL040W	GLK1	2.2	Glucokinase
YFR015C	GSY1	2.2	Glycogen synthase
YFR053C	HXK1	2.3	Hexokinase isoenzyme 1
YJL153C	INO1	3.6	L-myo-inositol-1-phosphate synthase

**Lipid, fatty acid and isoprenoid metabolism**			
YGL055W	OLE1	-2.3	Delta-9-fatty acid desaturase
YMR246W	FAA4	-1.9	Long chain fatty acyl-CoA synthetase
YBL039C	URA7	-1.7	Major CTP synthase isozyme
YLR056W	ERG3	-1.5	C-5 sterol desaturase
YDR096W	GIS1	1.4	JmjC domain-containing histone demethylase
YNL169C	PSD1	1.4	Phosphatidylserine decarboxylase of the mitochondrial inner membrane
YER026C	CHO1	1.6	Phosphatidylserine synthase
YBR183W	YPC1	1.8	Alkaline ceramidase
YJR073C	OPI3	2.4	Phospholipid methyltransferase
**RESPIRATION**			
YML120C	NDI1	1.4	NADH:ubiquinone oxidoreductase
YJR034W	PET191	1.4	Protein required for assembly of cytochrome c oxidase
YHR001W-a	QCR10	1.4	Subunit of the ubiqunol-cytochrome c oxidoreductase complex
YJL166W	QCR8	1.4	Subunit 8 of ubiquinol cytochrome-c reductase complex
YKL148C	SDH1	1.6	Flavoprotein subunit of succinate dehydrogenase
YDR231C	COX20	1.7	Mitochondrial inner membrane protein
YDL130W-a	STF1	1.8	Protein involved in regulation of the mitochondrial F1F0-ATP synthase
YGR008C	STF2	3.0	Protein involved in regulation of the mitochondrial F1F0-ATP synthase
**CELL CYCLE AND DNA PROCESSING**			
Cell Cycle			
YBR233W-a	DAD3	1.7	Essential subunit of the Dam1 complex
YDR022C	CIS1	1.5	Autophagy-specific protein required for autophagosome formation
YHR071W	PCL5	-2.2	Cyclin, interacts with and phosphorylated by Pho85p cyclin-dependent kinase (Cdk)
YJL141C	YAK1	1.4	Serine-threonine protein kinase
YKL161C	KDX1	1.6	Protein kinase implicated in the Slt2p mitogen-activated (MAP) kinase signaling pathway
**TRANSCRIPTION**			
RNA synthesis			
YBL054W	TOD6	-1.5	PAC motif binding protein involved in rRNA and ribosome biogenesis
YOR298C-a	MBF1	2.0	Transcriptional coactivator
YOR341W	RPA190	-1.5	RNA polymerase I largest subunit A190
YPR010C	RPA135	-1.4	RNA polymerase I second largest subunit A135
**RNA processing and modification**			
YDL048C	STP4	1.9	Protein containing a Kruppel-type zinc-finger domain
YML091C	RPM2	1.6	Protein subunit of mitochondrial RNase P

YPL123C	RNY1	1.6	Vacuolar RNase of the T(2) family
YNL119W	NCS2	-1.5	Protein required for thiolation of the uridine at the wobble position of Lys(UUU) and Glu(UUC) tRNAs
YDL112W	TRM3	-1.5	2'-O-ribose methyltransferase
YNR015W	SMM1	-1.5	Dihydrouridine synthase
YOL124C	TRM11	-1.6	Catalytic subunit of an adoMet-dependent tRNA methyltransferase complex
YPL207W	TYW1	2.0	Protein requir Protein required for the synthesis of wybutosine
**PROTEIN SYNTHESIS**			
**Ribosome biogenesis and rRNA processing**			
YAL059W	ECM1	-1.5	Pre-ribosomal factor involved in 60S ribosomal protein subunit export
YBR142W	MAK5	-1.7	Essential nucleolar protein
YDL031W	DBP10	-1.8	Putative ATP-dependent RNA helicase of the DEAD-box protein family
YDL060W	TSR1	-1.5	Protein required for processing of 20S pre-rRNA in the cytoplasm
YDL148C	NOP14	-1.4	Nucleolar protein
YDR060W	MAK21	-1.5	Constituent of 66S pre-ribosomal particles
YDR101C	ARX1	-1.5	Shuttling pre-60S factor
YER006W	NUG1	-1.4	GTPase that associates with nuclear 60S pre-ribosomes
YGL029W	CGR1	-1.6	Protein involved in nucleolar integrity and processing of the pre-rRNA for the 60S ribosome subunit
YGL078C	DBP3	-1.5	Putative ATP-dependent RNA helicase
YGR103W	NOP7	-1.7	Component of several different pre-ribosomal particles
YGR159C	NSR1	-1.4	Nucleolar protein that binds nuclear localization sequences
YHR066W	SSF1	-1.5	Constituent of 66S pre-ribosomal particles
YHR197W	RIX1	-1.7	Essential component of the Rix1 complex
YKL078W	DHR2	-1.7	Predominantly nucleolar DEAH-box ATP-dependent RNA helicase
YKR081C	RPF2	-1.7	Essential protein involved in the processing of pre-rRNA and the assembly of the 60S ribosomal subu
YMR128W	ECM16	-1.8	Essential DEAH-box ATP-dependent RNA helicase
YNL002C	RLP7	-1.4	Nucleolar protein with similarity to large ribosomal subunit L7 proteins
YOR294W	RRS1	-1.7	Protein that binds ribosomal protein L11 and is required for nuclear export of the 60S pre-ribosomal su
YPL012W	RRP12	-1.8	Protein required for export of the ribosomal subunits
YPL093W	NOG1	-1.7	Putative GTPase that associates with free 60S ribosomal subunits in the nucleolus
YOR056C	NOB1	-1.5	Essential nuclear protein involved in proteasome maturation and synthesis of 40S ribosomal subunits
YHR052W	CIC1	-1.7	Essential protein that interacts with proteasome components
YPL217C	BMS1	-1.8	GTPase required for synthesis of 40S ribosomal subunits and for processing the 35S pre-rRNA
YLR336C	SGD1	-1.5	Essential nuclear protein, required for biogenesis of the small ribosomal subunit
YOL041C	NOP12	-1.4	Nucleolar protein involved in pre-25S rRNA processing and biogenesis of large 60S ribosomal subunit
YMR269W	TMA23	-1.8	Nucleolar protein implicated in ribosome biogenesis
YKL082C	RRP14	-1.4	Essential protein, constituent of 66S pre-ribosomal particles

YLR196W	PWP1	-1.7	Protein involved in rRNA processing
YBL004W	UTP20	-1.6	Component of the small-subunit (SSU) processome
YBR247C	ENP1	-1.8	Protein associated with U3 and U14 snoRNAs
YCL054W	SPB1	-1.6	AdoMet-dependent methyltransferase involved in rRNA processing and 60S ribosomal subunit matura
YCR057C	PWP2	-1.6	Conserved 90S pre-ribosomal component essential for proper endonucleolytic cleavage of the 35 S rR
YDL153C	SAS10	-1.6	Essential subunit of U3-containing Small Subunit (SSU) processome complex
YGR128C	UTP8	-1.5	Nucleolar protein required for export of tRNAs from the nucleus
YGR145W	ENP2	-1.7	Essential nucleolar protein, required for biogenesis of the small ribosomal subunit
YGR280C	PXR1	-1.6	Essential protein involved in rRNA and snoRNA maturation
YIL019W	FAF1	-1.6	Protein required for pre-rRNA processing and 40S ribosomal subunit assembly
YJL033W	HCA4	-2.0	Putative nucleolar DEAD box RNA helicase
YJL050W	MTR4	-1.6	ATP-dependent 3'-5' RNA helicase of the Dead-box family
YLR002C	NOC3	-1.6	Protein that forms a nuclear complex with Noc2p
YLR129W	DIP2	-1.7	Nucleolar protein, specifically associated with the U3 snoRNA
YMR229C	RRP5	-1.5	RNA binding protein
YMR290C	HAS1	-1.5	ATP-dependent RNA helicase
YPL043W	NOP4	-1.6	Nucleolar protein, essential for processing and maturation of 27S pre-rRNA and large ribosomal subu
YPL266W	DIM1	-1.5	Essential 18S rRNA dimethylase (dimethyladenosine transferase)
YPR112C	MRD1	-1.9	Essential conserved protein that is part of the 90S preribosome
YGL120C	PRP43	-1.6	RNA helicase in the DEAH-box family
**PROTEIN FATE (folding, modification, destination)**			
**Protein/peptide degradation**			
YEL012W	UBC8	1.8	Ubiquitin-conjugating enzyme that negatively regulates gluconeogenesis
YHR138C		1.9	Putative protein of unknown function
YHR171W	ATG7	1.5	Autophagy-related protein and dual specificity member of the E1 family of ubiquitin-activating enzyme
YBL101C	ECM21	1.5	Protein involved in regulating the endocytosis of plasma membrane proteins
YPL003W	ULA1	1.5	Protein that acts together with Uba3p to activate Rub1p before its conjugation to proteins
YJL048C	UBX6	1.7	UBX (ubiquitin regulatory X) domain-containing protein that interacts with Cdc48p
**TRANSPORT**			
**Protein transport**			
YGR142W	BTN2	2.3	v-SNARE binding protein that facilitates specific protein retrieval from a late endosome to the Golgi
YNL036W	NCE103	1.7	Carbonic anhydrase
**Transmembrane transport**			
YDL054C	MCH1	-1.6	Protein with similarity to mammalian monocarboxylate permeases
YIL121W	QDR2	1.7	Multidrug transporter of the major facilitator superfamily
YDR497C	ITR1	1.7	Myo-inositol transporter

YKR039W	GAP1	1.9	General amino acid permeaseulated by nitrogen source
YBR132C	AGP2	2.1	High affinity polyamine permease
YMR011W	HXT2	-1.6	High-affinity glucose transporter of the major facilitator superfamily
**Mithocondral transport**			
YBR104W	YMC2	-1.5	Putative mitochondrial inner membrane transporter with a role in oleate metabolism and glutamate bic
**CELLULAR COMMUNICATION/SIGNAL TRANSDUCTION MECHANISM**			
Cellular signalling			
YGR136W	LSB1	1.5	Protein containing an N-terminal SH3 domain
YJL164C	TPK1	2.1	cAMP-dependent protein kinase catalytic subunit
YOL016C	CMK2	1.6	Calmodulin-dependent protein kinase
**CELL RESCUE, DEFENSE AND VIRULENCE**			
**Stress response**			
YBR155W	CNS1	-1.6	TPR-containing co-chaperone
YBR169C	SSE2	1.7	Member of the heat shock protein 70 (HSP70) family
YDL022W	GPD1	1.8	NAD-dependent glycerol-3-phosphate dehydrogenase
YDR171W	HSP42	2.8	Small heat shock protein (sHSP) with chaperone activity
YDR258C	HSP78	2.0	Oligomeric mitochondrial matrix chaperone
YDR513W	GRX2	1.4	Cytoplasmic glutaredoxin
YGR088W	CTT1	2.5	Cytosolic catalase T
YHR030C	SLT2	1.5	Serine/threonine MAP kinase
YIL113W	SDP1	1.5	Stress-inducible dual-specificity MAP kinase phosphatase,
YKL062W	MSN4	1.5	Transcriptional activator related to Msn2p
YKR092C	SRP40	-1.5	Nucleolar, protein that may function as a chaperone of small nucleolar ribonucleoprotein particles
YML100W	TSL1	3.0	Large subunit of trehalose 6-phosphate synthase (Tps1p)/phosphatase (Tps2p) complex
YNL007C	SIS1	1.8	Type II HSP40 co-chaperone that interacts with the HSP70 protein Ssa1p
YNL160W	YGP1	2.0	Cell wall-related secretory glycoprotein
YOL052C-a	DDR2	3.7	Multistress response protein
YPL004C	LSP1	1.6	Primary component of eisosomes
**INTERACTION WITH THE ENVIRONMENT**			
**Metal homeostasis**			
**Iron metabolism**			
YER174C	GRX4	3.7	Monothiol glutaredoxin
YER145C	FTR1	-3.3	High affinity iron permease
YLL027W	ISA1	1.5	Protein involved in biogenesis of iron-sulfur protein assembly machinery
YLR034C	SMF3	-1.4	NRAMP homolog Fe transporter
YLR220W	CCC1	2.7	Transporter that mediates vacuolar Fe storage

YMR058W	FET3	-6.9	Multicopper oxidase required for high-affinity Fe uptake
YNL240C	NAR1	-1.4	Component ofthe cytosolic iron-sulfur protein assembly machinery
**Copper metabolism**			
YPR124W	CTR1	-1.5	High-affinity copper transporter of the plasma membrane
YHR053C	CUP1-1	3.9	Copper binding metallothionein
YHR055C	CUP1-2	4.1	Copper binding metallothionein
**Zinc metabolism**			
YOL101C	IZH4	-1.9	Membrane protein involved in zinc ion homeostasis
**Calcium homeostasis**			
YGL006W	PMC1	1.5	Vacuolar Ca2+ ATPase involved in depleting cytosol of Ca2+ ions
**BIOGENESIS OF CELLULAR COMPONENTS**			
**Cell wall**			
YDL222C	FMP45	1.7	Integral membrane protein localized to mitochondria
YHR142W	CHS7	1.4	Protein involved in chitin biosynthesis by regulating Chs3p export from the ER
YJR106W	ECM27	1.5	Putative protein that may play a role in cell wall biosynthesis
YKL096W	CWP1	1.9	Cell wall mannoprotein
**UNKNOWN FUNCTION**			
YKR046C	PET10	1.4	Putative protein of unknown function
YIR030C	DCG1	1.9	Putative protein of unknown function
YLR327C	TMA10	2.2	Putative protein of unknown function
YBR054W	YRO2	1.6	Putative protein of unknown function
YAR027W	UIP3	1.6	Putative protein of unknown function
YBL029C-a		1.4	Putative protein of unknown function
YCR016W		-1.5	Putative protein of unknown function
YDL110C	TMA17	1.8	Putative protein of unknown function
YDL159W-a		1.8	Putative protein of unknown function
YDL204W	RTN2	2.3	Putative protein of unknown function
YDR379C-a		1.6	Putative protein of unknown function
YDR391C		1.5	Putative protein of unknown function
YER067W	RGI1	1.9	Putative protein of unknown function
YER137C		-1.6	Putative protein of unknown function
YHL021C	AIM17	2.1	Putative protein of unknown function
YHR087W	RTC3	3.2	Putative protein of unknown function
YIL024C		1.5	Putative protein of unknown function
YIL096C		-1.4	Putative protein of unknown function
YJL016W		1.4	Putative protein of unknown function

YJL163C		1.6	Putative protein of unknown function
YJR008W		1.7	Putative protein of unknown function
YKL091C		1.6	Putative protein of unknown function
YLR363W-a		-1.6	Putative protein of unknown function
YLR413W		-1.9	Putative protein of unknown function
YPL068C		-1.4	Putative protein of unknown function
YPR158W	CUR1	2.0	Putative protein of unknown function
**OTHERS OF KNOWN FUNCTION**			
YDL021W	GPM2	1.6	Homolog of Gpm1p phosphoglycerate mutase
YLR063W		-1.5	Putative S-adenosylmethionine-dependent methyltransferase
YER037W	PHM8	1.7	Lysophosphatidic acid (LPA) phosphatase
YNL124W	NAF1	-1.6	RNA-binding protein
YHR146W	CRP1	-1.4	Protein that binds to cruciform DNA structures
YJR004C	SAG1	1.4	Alpha-agglutinin of alpha-cells
YGL037C	PNC1	2.0	Nicotinamidase that converts nicotinamide to nicotinic acid
YGL053W	PRM8	1.7	Pheromone-regulated protein
YAL019W	FUN30	-1.5	Conserved member of the Snf2p family with ATP-dependent chromatin remodeling activity
YDL239C	ADY3	1.5	Protein required for spore wall formation
YDR085C	AFR1	1.8	Protein required for pheromone-induced projection (shmoo) formation
YDR516C	EMI2	1.8	Non-essential protein required for transcriptional induction of the early meiotic-specific transcription fa
YMR014W	BUD22	-1.7	Protein required for 18S rRNA maturation and small ribosomal subunit biogenesis
YDR361C	BCP1	-1.6	Essential protein involved in nuclear export of Mss4p
YOR173W	DCS2	2.0	Non-essential, stress induced regulatory protein
YPR175W	DPB2	-1.5	Second largest subunit of DNA polymerase II (DNA polymerase epsilon)
YGR248W	SOL4	2.1	6-phosphogluconolactonase with similarity to Sol3p
