# Supplementary material for: The Role of the Yap5 Transcription Factor in Remodeling Gene Expression in Response to Fe Bioavailability
Source: PLoS One. 2012 May 16;7(5):e37434. doi: 10.1371/journal.pone.0037434 (PMC3353947; doi:10.1371/journal.pone.0037434)
Supplement: Table S2 — Genes dependent on Yap5 whose mRNA steady-state levels are altered upon cells incubation with 2 mM of FeSO4 for 20 min. (PDF) [file pone.0037434.s002.pdf]

Table S2. Genes dependent on Yap5 whose steady-state levels are altered upon cells incubation with2mM of FeSO4 for 20 min

Systematic Name	Gene Name	Fold Change	Description
**CELL CYCLE AND DNA PROCESSING**			
**DNA processing**			
YLR103c	CDC45	-1.5	DNA replication initiation factor
YDR386w	MUS81	-1.4	Subunit of the structure-specific Mms4p-Mus81p endonuclease that cleaves branched DNA
YOL017w	ESC8	-1.4	Protein involved in telomeric and mating-type locus silencing
YEL019c	MMS21	-1.4	Protein involved in DNA repair
YER116c	SLX8	1.4	Subunit of the Slx5-Slx8 SUMO-targeted ubiquitin ligase (STUbL) complex
YGL113w	SLD3	1.5	Protein involved in the initiation of DNA replication
YFR023w	PES4	1.5	Poly(A) binding protein
Cell cycle			
YDR168w	CDC37	-1.4	Essential Hsp90p co-chaperone
YLR079w	SIC1	1.6	Inhibitor of Cdc28-Clb kinase complexes that controls G1/S phase transition
YOR195w	SLK19	1.7	Kinetochore-associated protein
**TRANSCRIPTION**			
RNA synthesis			
YGR097w	ASK10	1.6	Component of RNA polymerase II holoenzyme
YGL043w	DST1	-1.4	General transcription elongation factor TFIIS
YOL042w	NGL1	-1.4	Putative endonuclease
**RNA processing**			
YCR073w-a	SOL2	1.6	Protein with a possible role in tRNA export
YNL282w	POP3	1.5	Subunit of both RNase MRP and nuclear RNase P
**PROTEIN SYNTHESIS**			
YBL080c	PET112	-1.8	Subunit of the trimeric GatFAB AmidoTransferase(AdT) complex
YLR067c	PET309	-1.6	Specific translational activator for the COX1 mRNA
YLR289w	GUF1	-1.5	Mitochondrial matrix GTPase
**PROTEIN FATE (folding, modification, destination)**			
**Protein/peptide degradation**			
YMR119w	ASI1	-1.7	Putative integral membrane E3 ubiquitin ligase
YJR062c	NTA1	1.5	Amidase
YIL030c	SSM4	2.1	Ubiquitin-protein ligase involved in ER-associated protein degradation
**CELL RESCUE, DEFENSE AND VIRULENCE**			

**Stress response**			
YMR140w	SIP5	-1.7	Protein of unknown function
YOR386w	PHR1	-1.5	DNA photolyase involved in photoreactivation
YER118c	SHO1	1.5	Transmembrane osmosensor
YFL016c	MDJ1	1.6	Co-chaperone
**NTERACTION WITH THE ENVIRONMENT**			
**Metal homeostasis**			
YLR220w	CCC1	-2.9	Transporter that mediates vacuolar Fe storage
YER174c	GRX4	-2.7	Monothiol glutaredoxin
**UNKNOWN FUNCTION**			
YGR126w		-1.5	Putative protein of unknown function
YHR131c		1.4	Putative protein of unknown function
YNL295w		1.4	Putative protein of unknown function
YOR019w		1.4	Protein of unknown function that may interact with ribosomes
YLR346c		1.5	Putative protein of unknown function found in mitochondria
YBR032w		1.5	Dubious open reading frame
YGR168c		1.5	Putative protein of unknown function
YFL067w		1.5	Protein of unknown function, down-regulated at low calcium levels
YJL055w		1.6	Putative protein of unknown function
YBR150c	TBS1	1.8	Putative protein of unknown function
YLR190w	MMR1	1.9	Phosphorylated protein of the mitochondrial outer membrane,
YDR381c-a		2.2	Protein of unknown function, localized to the mitochondrial outer membrane
YER007c-a	TMA20	1.6	Protein of unknown function that associates with ribosomes and has a putative RNA binding domain
**OTHERS OF KNOWN FUNCTION**			
YNR059w	MNT4	-1.7	Putative alpha-1,3-mannosyltransferase
YIL082w-a		1.4	Retrotransposon TYA Gag and TYB Pol genes
YBL088c	TEL1	1.5	Protein kinase primarily involved in telomere length regulation
YDL214c	PRR2	1.5	Serine/threonine protein kinase that inhibits pheromone induced signalling downstream of MAPK
YOR255w	OSW1	1.5	Protein involved in sporulation
**amino acid metabolism**			
YOL058w	ARG1	-1.6	Arginosuccinate synthetase
**nucleotide/nucleoside/nucleobase metabolism**			
YMR271c	URA10	-1.5	Minor orotate phosphoribosyltransferase (OPRTase) isozyme
**C-compound and carbohydrate metabolism**			
YJL153c	INO1	-2.9	L-myo-inositol-1-phosphate synthase
**lipid, fatty acid and isoprenoid metabolism**			

YPL148c	PPT2	1.7	Phosphopantetheine:protein transferase (PPTase)
YBL042c	FUI1	1.5	High affinity uridine permease
YER105c	NUP157	1.8	Abundant subunit of the nuclear pore complex (NPC)
