# Supplementary material for: The Role of the Yap5 Transcription Factor in Remodeling Gene Expression in Response to Fe Bioavailability
Source: PLoS One. 2012 May 16;7(5):e37434. doi: 10.1371/journal.pone.0037434 (PMC3353947; doi:10.1371/journal.pone.0037434)
Supplement: Table S3 — Genes dependent on Yap5 whose mRNA steady-state levels are altered upon cells incubation with2 mM of FeSO4 for 60 min. (PDF) [file pone.0037434.s003.pdf]

Table S3. Genes dependent on Yap5 whose steady-state levels are altered upon cells incubation with2mM of FeSO4 for 60 min

Systematic Name	Gene Name	Fold Change	Description
**METABOLISM**			
**C-Compound and Carbohydrate metabolism**			
YGR087C	PDC6	-2.7	Pyruvate decarboxylase isozyme
YOR178C	GAC1	-2.1	Regulatory subunit for Glc7p type-1 protein phosphatase
YJL153C	INO1	-1.7	L-myo-inositol-1-phosphate synthase
YGL256W	ADH4	1.5	Alcohol dehydrogenase isoenzyme IV
YGL035C	MIG1	1.5	Transcription factor involved in glucose repression
**Lipid, fatty acid and isoprenoid metabolism**			
YBR058C-A	TSC3	-2.4	Protein involved in sphingolipid biosynthesis
YMR101C	SRT1	-2.3	Cis-prenyltransferase involved in synthesis of long-chain dolichols
YDR018C		-1.8	Protein similar to 1-acyl-glycerol-3-phosphate acyltransferase
YDR287W	INM2	-1.8	Inositol monophosphatase
YGL055W	OLE1	-1.7	Delta-9-fatty acid desaturase
YGL205W	POX1	-1.5	Fatty-acyl coenzyme A oxidase
YBR042C	CST26	1.4	Protein required for incorporation of stearic acid into phosphatidylinositol
YJL091C	GWT1	1.6	Protein involved in the inositol acylation of glucosaminyl phosphatidylinositol
**RESPIRATION**			
Q0275	COX3	-3.5	Cytochrome c oxidase subunit III
Q0050	AI1	-2.3	Reverse transcriptase required for splicing of the COX1 pre-mRNA
Q0080	ATP8	-2.2	ATP synthase subunit 8
YJL045W		-2.2	Minor succinate dehydrogenase isozyme
YLR201C	COQ9	1.4	Protein required for ubiquinone biosynthesis and respiratory growth
YML129C	COX14	1.4	Protei involved in translational regulation of Cox1p and assembly of cytochrome c oxidase
**CELL CYCLE AND DNA PROCESSING**			
**DNA processing**			
YBR098W	MMS4	-2.3	Endonuclease that cleaves branched DNA
YEL019C	MMS21	-1.8	Protein involved in DNA repair
YPR164W	MMS1	-1.6	Subunit of an E3 ubiquitin ligase complex
YBR060C	ORC2	1.4	Subunit of the origin recognition complex
YLR247C	IRC20	1.4	Putative helicase
YGL113W	SLD3	1.5	Protein involved in the initiation of DNA replication

YLR135W	SLX4	1.6	Endonuclease involved in processing DNA during recombination and repair
YML062C	MFT1	1.7	Subunit of the THO complex
**Cell Cycle**			
YKL089W	MIF2	-3.1	Kinetochore protein with homology to human
YER111C	SWI4	-2.6	DNA binding component of the SBF complex (Swi4p-Swi6p)
YBR107C	IML3	-2.6	Protein with a role in kinetochore function
YBL097W	BRN1	-2.1	Subunit of the condensin complex
YER018C	SPC25	-2.0	Component of the kinetochore-associated Ndc80 complex
YER106W	MAM1	-2.0	Monopolin
YDR446W	ECM11	-2.0	Non-essential protein apparently involved in meiosis
YIL073C	SPO22	-1.9	Meiosis-specific protein essential for chromosome synapsis
YOR349W	CIN1	-1.8	Tubulin folding factor D involved in beta-tubulin folding
YNL188W	KAR1	-1.7	Essential protein involved in spindle pole body duplication during mitosis
YBR186W	PCH2	-1.6	Nucleolar component of the pachytene checkpoint
YPL121C	MEI5	-1.6	Meiosis specific protein
YIR025W	MND2	-1.6	Subunit of the anaphase-promoting complex (APC)
YLR329W	REC102	-1.6	Protein involved in early stages of meiotic recombination
YOR177C	MPC54	-1.6	Component of the meiotic outer plaque
YFR027W	ECO1	-1.4	Acetyltransferase required for establishment of sister chromatid cohesion
YDL008W	APC11	1.4	Catalytic core subunit of the Anaphase-Promoting Complex/Cyclosome (APC/C)
YIL144W	TID3	1.5	Component of the kinetochore-associated Ndc80 complex
YML104C	MDM1	1.6	Intermediate filament protein
YBR215W	HPC2	1.7	Subunit of the HIR nucleosome assembly complex
YLR212C	TUB4	1.8	Gamma tubulin
YJL106W	IME2	2.1	erine/threonine protein kinase involved in activation of meiosis
**TRANSCRIPTION**			
**RNA processing**			
YKL095W	YJU2	-3.3	Essential protein required for pre-mRNA splicing
YKR060W	UTP30	-3.0	Subunit of U3-containing 90S preribosome complex
Q0255		-2.3	Maturase-like protein
YBL074C	AAR2	-2.2	Component of the U5 snRNP
Q0120	BI4	-2.0	Mitochondrial mRNA maturase
YNL282W	POP3	-1.6	Subunit of both RNase MRP and nuclear RNase P
YPR072W	NOT5	1.4	Subunit of the CCR4-NOT complex
YPR042C	PUF2	1.5	mRNA binding protein
YJL203W	PRP21	1.6	Subunit of the SF3a splicing factor complex

YFR005C	SAD1	1.7	Conserved zinc-finger domain protein involved in pre-mRNA splicing
YKR002W	PAP1	1.7	Poly(A) polymerase
YHL038C	CBP2	2.0	Mitochondrial protein required for splicing of the group I intron aI5 of the COB pre-mRNA
**PROTEIN FATE (folding, modifiCation, destination)**			
**Protein/peptide degradation**			
YOL141W	PPM2	-3.6	tRNA methyltransferase also involved in methoxycarbonylation and equired for the synthesis of wybutc
YHR160C	PEX18	-2.9	Peroxin required for targeting of peroxisomal matrix proteins containing PTS2
YKL171W	NNK1	-2.0	Protein kinase implicated in proteasome function
YDL008W	APC11	1.4	Catalytic core subunit of the Anaphase-Promoting Complex/Cyclosome (APC/C)
YDR425W	SNX41	1.5	Sorting nexin
YOR297C	TIM18	1.6	Component of the mitochondrial TIM22 complex
YFR003C	YPI1	1.6	Inhibitor of the type I protein phosphatase Glc7p
YOL013C	HRD1	1.6	Ubiquitin-protein ligase required for endoplasmic reticulum-associated degradation
YDR069C	DOA4	1.9	Ubiquitin isopeptidase
**TRANSPORT**			
YNL253W	TEX1	-2.9	Protein involved in mRNA export
YCR010C	ADY2	-2.4	Acetate transporter required for normal sporulation
YAL067C	SEO1	-2.4	Putative permease
YNL318C	HXT14	-2.0	Protein with similarity to hexose transporter family members
YFL011W	HXT10	-1.6	Putative hexose transporter
YLL052C	AQY2	-1.6	Water channel that mediates the transport of water across cell membranes
YGR009C	SEC9	1.4	t-SNARE protein important for fusion of secretory vesicles with the plasma membrane
YER119C	AVT6	1.4	Vacuolar aspartate and glutamate exporte
YNL051W	COG5	1.5	Component of the oligomeric Golgi complex that functions in protein trafficking
YBR080C	SEC18	1.5	ATPase required for vesicular transport between ER and Golgi
YPL265W	DIP5	1.5	Dicarboxylic amino acid permease
YIL114C	POR2	1.6	Putative mitochondrial porin
YJR005W	APL1	1.7	Beta-adaptin involved in vesicle mediated transport;
YDR406W	PDR15	2.1	Plasma membrane ATP binding cassette (ABC) transporter, multidrug transporter
**CELL RESCUE, DEFENSE AND VIRULENCE**			
**Stress response**			
YFL014W	HSP12	-1.4	Plasma membrane protein involved in maintaining membrane organization in stress conditions
YFL031W	HAC1	-1.4	Transcription factor that regulates the unfolded protein response
YIL113W	SDP1	-1.5	Stress-inducible dual-specificity MAP kinase phosphatase,
YJL144W		-3.4	Cytoplasmic hydrophilin with a role in dessication resistance
YGR154C	GTO1	-2.4	Omega-class glutathione transferase

YOR178C	GAC1	-2.1	Regulatory subunit for Glc7p type-1 protein phosphatase
YPL280W	HSP32	-1.6	Possible chaperone and cysteine protease
Detoxification			
YGR138C	TPO2	-1.5	Polyamine transport protein
YPR156C	TPO3	-1.4	Polyamine transport protein
YBR008C	FLR1	1.4	Plasma membrane multidrug transporter of the major facilitator superfamily
YOR273C	TPO4	1.6	Polyamine transport protein
**INTERACTION WITH THE ENVIRONMENT**			
**Metal homeostasis**			
YOL152W	FRE7	-2.6	Putative ferric reductase with similarity to Fre2p with expression induced by low copper levels
YER174C	GRX4	-1.9	Monothiol glutaredoxin
YLR220W	CCC1	-1.7	Transporter that mediates vacuolar Fe storage
YDR205W	MSC2	1.4	Member of the cation diffusion facilitator family
YPL207W	TYW1	-1.5	Protein required for the synthesis of wybutosine
YCR044C	PER1	1.5	Protein of the endoplasmic reticulum
YDR182W	CDC1	1.5	Putative lipid phosphatase of the endoplasmic reticulum
YMR058W	FET3	2.4	Multicopper oxidase required for high-affinity Fe uptake
**UNKNOWN FUNCTION**			
YLR162W		-4.8	Putative protein of unknown function
YAR066W		-3.8	Putative GPI protein
YBL059W		-3.5	Putative protein of unknown function
YHR112C		-3.3	Putative protein of unknown function
YPL077C		-3.3	Putative protein of unknown function
YMR084W		-2.7	Putative protein of unknown function
YMR317W		-2.7	Protein of unknown function
YGR035C		-2.7	Putative protein of unknown function
YJL132W		-2.6	Putative protein of unknown function
YEL008W		-2.5	Hypothetical protein
YLR161W		-2.2	Putative protein of unknown function
YNL195C		-2.2	Putative protein of unknown function
YBR076W	ECM8	-2.1	Protein of unknown function
YLR281C		-2.1	Putative protein of unknown function
YLR281C		-2.1	Protein of unknown function
YAL064C-A		-2.1	Putative protein of unknown function
YPL205C		-2.1	Hypothetical protein
YJR120W		-2.1	Protein of unknown function

YGR126W		-2.1	Putative protein of unknown function
YJL218W		-2.0	Putative protein of unknown function
YPL165C	SET6	-2.0	Protein of unknown function
YGL258W-A		-1.9	Putative protein of unknown function
YJR108W	ABM1	-1.9	Protein of unknown function
YMR265C		-1.8	Putative protein of unknown function
YBR033W	EDS1	-1.8	Putative zinc cluster protein
YPR116W	RRG8	-1.7	Putative protein of unknown function
YHR177W		-1.7	Putative protein of unknown function
YJL105W	SET4	-1.7	Protein of unknown function
YMR018W		-1.7	Putative protein of unknown function
YJL038C	LOH1	-1.6	Putative protein of unknown function
YNL095C		-1.5	Putative protein of unknown function
YGL117W		-1.5	Putative protein of unknown function
YHL045W		-1.5	Putative protein of unknown function
YCL058C	FYV5	-1.4	Protein of unknown function
YHR126C	ANS1	-1.4	Putative protein of unknown function
YJL131C	AIM23	1.4	Putative protein of unknown function
YML131W		1.4	Putative protein of unknown function
YHR078W		1.4	High osmolarity-regulated gene of unknown function
YKR021W	ALY1	1.5	Putative protein of unknown function
YIL090W	ICE2	1.5	Integral ER membrane protein
YJL078C	PRY3	1.5	Protein of unknown function
YDR111C	ALT2	1.5	Putative alanine transaminase
YGL185C		1.5	Putative protein of unknown function
YPL260W		1.5	Protein of unknown function
YDR475C	JIP4	1.5	Protein of unknown function
YOL013W-A		1.6	Protein of unknown function
YCR061W		1.6	Protein of unknown function
YGL226W	MTC3	1.6	Protein of unknown function
YDR068W	DOS2	1.6	Protein of unknown function
YIL096C		1.6	Putative protein of unknown function
**OTHERS OF KNOWN FUNCTION**			
YMR025W	CSI1	-3.2	Subunit of the Cop9 signalosome
YDR210W-A		-2.9	Retrotransposon TYA Gag gene
YNL230C	ELA1	-2.8	Elongin A, protein required for ubiquitin-dependent degredation of the RNA Polymerase II subunit RPC

YOR134W	BAG7	-2.4	Rho GTPase activating protein
YBR240C	THI2	-2.3	Transcriptional activator of thiamine biosynthetic genes
YAL062W	GDH3	-2.3	NADP(+)-dependent glutamate dehydrogenase
YDR170W-A		-2.3	Retrotransposon TYA Gag gene
YOR009W	TIR4	-2.2	Cell wall mannoprotein
YOL164W	BDS1	-2.2	Bacterially-derived sulfatase
YPL033C	SRL4	-2.2	Protein involved in regulation of dNTP production
YPL192C	PRM3	-2.2	Pheromone-regulated protein required
YJL170C	ASG7	-2.1	An a-specific gene that is induced by alpha factor
YIL037C	PRM2	-2.1	Pheromone-regulated protein
YPL074W	YTA6	-2.1	Putative ATPase
YLL046C	RNP1	-2.0	Ribonucleoprotein that contains two RNA recognition motifs
YMR107W	SPG4	-2.0	Protein required for survival at high temperature during stationary phase
YOR068C	VAM10	-1.9	Protein involved in vacuole morphogenesis
YOR358W	HAP5	-1.9	Subunit of the heme-activated, glucose-repressed Hap2/3/4/5 CCAAT-binding complex,
YNL194C		-1.9	Integral membrane protein required for sporulation and plasma membrane sphingolipid content
YKL132C	RMA1	-1.9	Putative dihydrofolate synthetase
YPL027W	SMA1	-1.8	Protein of unknown function involved in the assembly of the prospore membrane during sporulation
YJL114W		-1.8	Retrotransposon TYA Gag gene
YLR453C	RIF2	-1.6	Protein that helps to control telomere length and to establish telomeric silencing
YDR403W	DIT1	-1.6	Sporulation-specific enzyme required for spore wall maturation
YPL268W	PLC1	-1.5	Phospholipase C
YOR076C	SKI7	-1.5	Coupling protein that mediates 3'-5' RNA degradation
YDR210C-C		-1.5	Retrotransposon TYA Gag gene
YDR261C-C		-1.4	Retrotransposon TYA Gag gene
YOL103W-A		-1.4	Retrotransposon TYA Gag gene
YDR490C	PKH1	1.4	Serine/threonine protein kinase involved in endocytosisand cell wall integrity
YPL049C	DIG1	1.4	MAP kinase-responsive inhibitor of the Ste12p transcription factor
YCR019W	MAK32	1.4	Protein necessary for structural stability of L-A double-stranded RNA-containing particles
YGL208W	SIP2	1.5	One of three beta subunits of the Snf1 serine/threonine protein kinase complex
YGL107C	RMD9	1.5	Mitochondrial protein required for respiratory growth
YPL161C	BEM4	1.5	Protein involved in establishment of cell polarity and bud emergence
YNL081C	SWS2	1.5	Putative mitochondrial ribosomal protein of the small subunit
YBL103C	RTG3	1.6	Basic helix-loop-helix-leucine zipper (bHLH/Zip) transcription factor
YIL071C	PCI8	1.6	Protein with possible dual functions in transcriptional and translational control
YHR011W	DIA4	1.6	Probable mitochondrial seryl-tRNA synthetase

YGR215W	RSM27	1.7	Mitochondrial ribosomal protein of the small subunit
YDL240W	LRG1	1.7	Putative GTPase-activating protein involved in the controls cell wall integrit
YBR101C	FES1	1.7	Hsp70 nucleotide exchange factor
YLR363C	NMD4	1.8	Protein interacting with Nam7p
YDR264C	AKR1	1.8	Palmitoyl transferase involved in protein palmitoylation
YNR020C	ATP23	1.9	Putative metalloprotease of the mitochondrial inner membrane
YHR061C	GIC1	1.9	Protein of unknown function involved in initiation of budding and cellular polarization
