# Supplementary material for: The Role of the Yap5 Transcription Factor in Remodeling Gene Expression in Response to Fe Bioavailability
Source: PLoS One. 2012 May 16;7(5):e37434. doi: 10.1371/journal.pone.0037434 (PMC3353947; doi:10.1371/journal.pone.0037434)
Supplement: Figure S1 — Copper metabolism in yeast is affected upon growth shift to high-Fe medium. BY4742 wild-type cells were transformed with a plasmid containing CCS1 (copper chaperone for Cu/Zn superoxide dismutase) HA-tagged and exponentially grown in SD medium. (A) Cells were treated with 2 mM of FeSO4, harvested at the indicated time-points and examined by Western blot with an anti-HA antibody. (B) Ccs1-HA response to high-Cu (9 mM CuSO4, 60 min) was monitored by Western blot and served as Ccs1-HA functional control. Sba1 protein levels were used as loading control. (PDF) [file pone.0037434.s004.pdf]

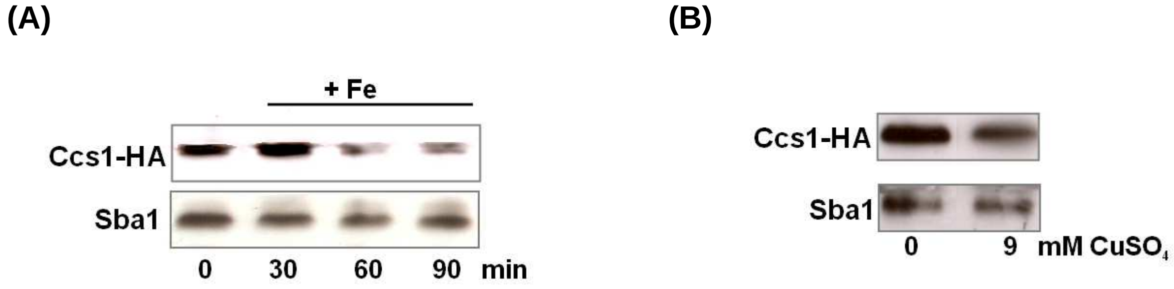

Figure S1. **Copper metabolism in yeast is affected upon growth shift to high-Fe medium.** BY4742 wild-type cells were transformed with a plasmid containing *CCS1* (copper chaperone for Cu/Zn superoxide dismutase) HA-tagged and exponentially grown in SD medium. (A) Cells were treated with 2mM of $FeSO_4$, harvested at the indicated time-points and examined by Western blot with an anti-HA antibody. (B) Ccs1-HA response to high-Cu (9mM $CuSO_4$, 60min) was monitored by Western blot and served as Ccs1-HA functional control. Sba1 protein levels were used as loading control.
