# Supplementary material for: The Role of the Yap5 Transcription Factor in Remodeling Gene Expression in Response to Fe Bioavailability
Source: PLoS One. 2012 May 16;7(5):e37434. doi: 10.1371/journal.pone.0037434 (PMC3353947; doi:10.1371/journal.pone.0037434)
Supplement: Figure S2 — In the absence of Ccc1, Yap1 is required for cells to overcome Fe-induced oxidative stress. Exponentially growing cells from wild-type (BY4742), yap1, yap1ccc1 and ccc1 strains were harvested, serially diluted and spotted onto control SC plates or SC plates containing the indicated FeSO4 concentrations under (A) aerobiosis and (B) anaerobiosis. (PDF) [file pone.0037434.s005.pdf]

(A)

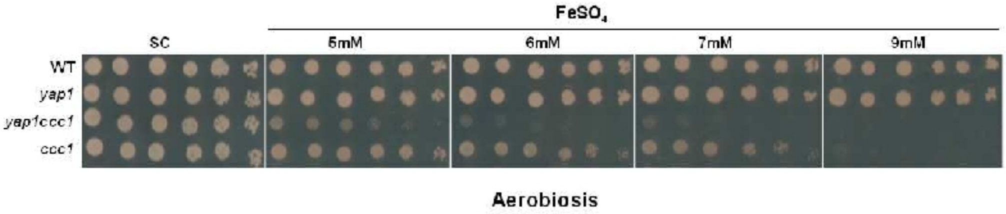

(B)

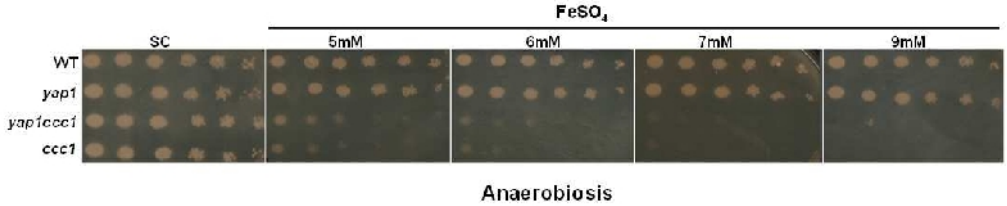

Figure S2. In the absence of Ccc1, Yap1 is required for cells to overcome Fe-induced oxidative stress. Exponentially growing cells from wild-type (BY4742), *yap1*, *yap1ccc1* and *ccc1* strains were harvested, serially diluted and spotted onto control SC plates or SC plates containing the indicated $FeSO_4$ concentrations under (A) aerobiosis and (B) anaerobiosis.
