# Supplementary material for: The Role of the Yap5 Transcription Factor in Remodeling Gene Expression in Response to Fe Bioavailability
Source: PLoS One. 2012 May 16;7(5):e37434. doi: 10.1371/journal.pone.0037434 (PMC3353947; doi:10.1371/journal.pone.0037434)
Supplement: Figure S3 — Yap5 transactivation potential in different media used in this work. (B) The transactivation potential of Yap5 in SD medium not supplemented (SD) or supplemented with 100 µM of BPS (SD-Fe), or 2 mM of FeSO4 (SD+Fe), was assayed. EGY48 strain carrying pSH18-34 (a plasmid carrying a lacZ reporter gene) was transformed with Yap5-LexA and ß-galactosidase activity was monitored as described in Experimental procedures. Values are the mean of triplicate samples of the same experiment ± s.d. (PDF) [file pone.0037434.s006.pdf]

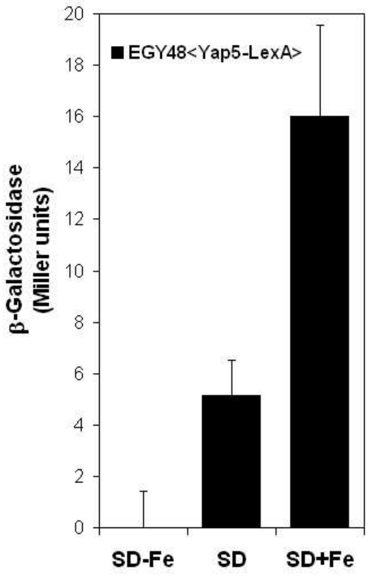

Figure S3. **Yap5 transactivation potential in different media used in this work.** The transactivation potential of Yap5 in SD medium not supplemented (SD) or supplemented with 100μM of BPS (SD-Fe), or 2mM of $FeSO_4$ (SD+Fe), was assayed. EGY48 strain carrying pSH18-34 (a plasmid carrying a *lacZ* reporter gene) was transformed with Yap5-LexA and β-galactosidase activity was monitored as described in *Experimental procedures*. Values are the mean of triplicate samples of the same experiment ± s.d.
